# Natural extracts, honey, and propolis as human norovirus inhibitors

**DOI:** 10.1038/s41598-022-11643-5

**Published:** 2022-05-17

**Authors:** Kerstin Ruoff, Jessica Michelle Devant, Grant Hansman

**Affiliations:** 1 Schaller Research Group at the University of Heidelberg and the DKFZ, Heidelberg, Germany; 2 Department of Infectious Diseases, Virology, University of Heidelberg, Heidelberg, Germany; 3 Present Address: Institute for Glycomics, Griffith University, Gold Coast Campus, Gold Coast, QLD Australia

**Keywords:** Microbiology, Infectious-disease diagnostics

## Abstract

Norovirus is the most important cause of acute gastroenteritis, yet there are still no antivirals, vaccines, or treatments available. Several studies have shown that norovirus-specific monoclonal antibodies, Nanobodies, and natural extracts might function as inhibitors. Therefore, the objective of this study was to determine the antiviral potential of additional natural extracts, honeys, and propolis samples. Norovirus GII.4 and GII.10 virus-like particles (VLPs) were treated with different natural samples and analyzed for their ability to block VLP binding to histo-blood group antigens (HBGAs), which are important norovirus co-factors. Of the 21 natural samples screened, date syrup and one propolis sample showed promising blocking potential. Dynamic light scattering indicated that VLPs treated with the date syrup and propolis caused particle aggregation, which was confirmed using electron microscopy. Several honey samples also showed weaker HBGA blocking potential. Taken together, our results found that natural samples might function as norovirus inhibitors.

## Introduction

Human noroviruses typically cause an acute illness of fever, nausea, vomiting, cramping, malaise, and diarrhea that can typically persists for 2 to 5 days. Noroviruses are highly contagious and 10–100 viral particles may be sufficient to infect an individual. The US Centers for Disease Control and Prevention estimates that it is the most common cause of acute gastroenteritis in the United States with 21 million cases each year and an estimated 70,000 hospitalizations and 800 deaths per year. Worldwide, norovirus infections account for ~ 20% of diarrheal illnesses and as many as 200,000 child deaths each year^1^, however the actual numbers of unreported cases are presumably much higher. Norovirus infections can occur in all age groups and results in significant morbidity and mortality, particularly in the very old and very young patients. Preventive measures against norovirus include hand washing, isolation of those infected, meticulous cleaning and disinfection.

Noroviruses have a single-stranded, positive sense RNA genome of ~ 7.4 to 7.7 kbases. The genome contains three open reading frames (ORFs), where ORF1 encodes the non-structural proteins, which include the protease and RNA dependent RNA polymerase (RdRp), ORF2 encodes the capsid protein (VP1), and ORF3 encodes a minor structural protein. Based on the capsid amino acid sequences, noroviruses can be divided into at least ten genogroups (GI-GX), with GI, GII, and GIV mainly causing infections in human^2^. Each of these genogroups is further subdivided into numerous genotypes. Human noroviruses often evolve into genetically and antigenically variant strains, and frequent genetic recombination at the RdRp and capsid junction increases the genetic and antigenic diversity.

Expression of the capsid gene in insect cells leads to the self-assembly of virus-like particles (VLPs) that are antigenically similar to the native virions with a T = 3 icosahedral symmetry. However, recent structural studies have discovered the sizes of VLPs can vary among the genotypes, where GII.4 VLPs were discovered to mainly fold into particles with T = 4 icosahedral symmetry, but also native sized T = 3 and smaller T = 1 particles were observed^3,4^. Likewise, the GII.10 VLPs exhibited both T = 3 and T = 1 icosahedral symmetry^5,6^. The X-ray crystal structure of norovirus VLPs identified two domains, shell (S) and protruding (P) domain. The S domain surrounds the viral RNA, whereas the P domain, which can be further subdivided into P1 and P2 subdomains, and contains determinants for co-factor attachment and antigenicity.

Noroviruses bind histo-blood group antigen (HBGA) co-factors, which are polymorphic carbohydrate structures present as free antigens in saliva and found on the surface of various epithelia. HBGAs are typically grouped into either ABH or Lewis types and at least nine different HBGA co-factors have been recognized to bind to noroviruses^7–13^. The ABH types are characterized by an α-l-fucose-(1-2)-β-d-galactose connection, whereas the Lewis types contain an α-l-fucose-(1-3)/(1-4)-β-d-*N*-acetyl-glucosamine. The GII noroviruses primarily interact with the ABH-fucose, Lewis-fucose, or a combination of both. Earlier structural studies showed that noroviruses bind two HBGA molecules per P domain dimer, and the GI and GII noroviruses bind HBGAs in different regions on the capsid. We recently identified two additional fucose-binding pockets on the GII norovirus capsid, which suggested that HBGA binding interactions are likely more complex than previously recognized^10,14,15^. Moreover, both epidemic and rarely detected noroviruses bind multiple HBGA types^10,11,16^.

The HBGA- and fucose-binding pockets on the norovirus capsid are one conceivable target for antiviral compounds. Indeed, we found several compounds that bind at the HBGA pocket, i.e., human milk oligosaccharides (HMOs), citrate, and molecules that overlap the HBGA pocket, i.e., norovirus-specific Nanobodies and a norovirus-specific monoclonal antibody^5,17–20^. A number of other studies have also discovered norovirus-specific monoclonal antibodies that can inhibit HBGA binding^21–25^. The identification of such compounds blocking HBGA binding are potential treatments for norovirus infections. A number of studies have also examined the HBGA blocking potential of natural extracts, for example Chinese gall, pomegranate juice, and tannic acid^26^, cranberry juice^27^, grape seed extract^28^, persimmon extract and tannins^29^, and green tea extracts^30^. Many of these extracts were found to target the HBGA pocket and show great promise as natural therapies.

In this study, we screened seven different natural extracts (coconut blossom syrup, date syrup, agave nectar, apple sweetener, royal jelly, barley malt, maple syrup), ten different honey samples, and four propolis samples to identify inhibitors that block norovirus GII.4 and GII.10 VLPs from binding to HBGAs. Our data showed that several natural samples (date syrup, honey, and propolis) inhibited VLPs from binding to HBGAs. Treatment of VLPs with these samples indicated a disruption of VLP integrity and resulted in aggregation of particles. Overall, our results indicated that both epidemic and rarely detected genotypes could be blocked from binding to HBGAs with date syrup and propolis.

## Results

### Natural extract treatment

**Figure 1:**
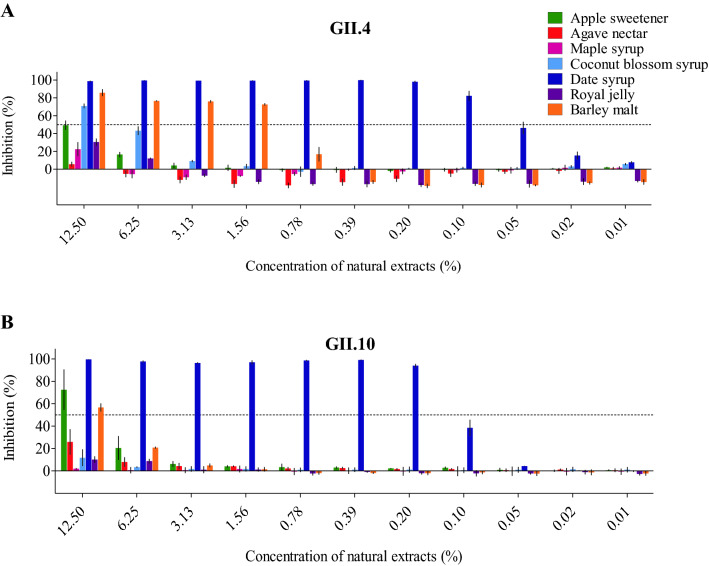
Natural extracts blocking GII.4 and GII.10 VLPs attachment to HBGAs. VLPs were mixed with serially diluted natural extracts and the HBGA attachment inhibition level was measured using ELISA. The final concentration of the natural extracts ranged between 12.5 and 0.01%. All experiments were performed in triplicates and the standard deviation is shown as error bars and the IC_50_ cutoff is indicated by a dashed line. (**A**) For GII.4 VLPs, date syrup showed the strongest inhibition (IC_50_ = 0.06%) followed by barley malt (IC_50_ = 0.9%). All other natural extracts showed little blocking potential at the concentrations tested. (**B**) For GII.10 VLPs, date syrup inhibited binding to HBGAs at a low concentration (IC_50_ = 0.11%), whereas other natural extracts were not effective at the concentrations tested.

Seven natural extracts were examined for their ability to block the epidemic GII.4 and the rarely detected GII.10 norovirus VLPs from binding to HBGAs using ELISA (Fig. 1). Date syrup blocked both GII.4 and GII.10 VLPs from HBGA binding at low concentrations (IC_50_ = 0.06% and 0.11%, respectively). Coconut blossom syrup and apple sweetener only weakly inhibited GII.4 VLPs (IC_50_ = 5.84% and 14.96%, respectively), but not GII.10 VLPs. These three extracts showed a dose-dependent inhibition. Several natural extracts exhibited unusual binding properties with the GII.4 VLPs, i.e., barley malt, royal jelly, and agave nectar showed negative-inhibition effects at low concentrations. At concentrations above 0.78%, barley malt inhibited GII.4 VLPs (IC_50_ = 0.90%). This unusual GII.4 inhibition event with barley malt was also observed with 2’FL inhibition assays (unpublished data).

**Figure 2:**
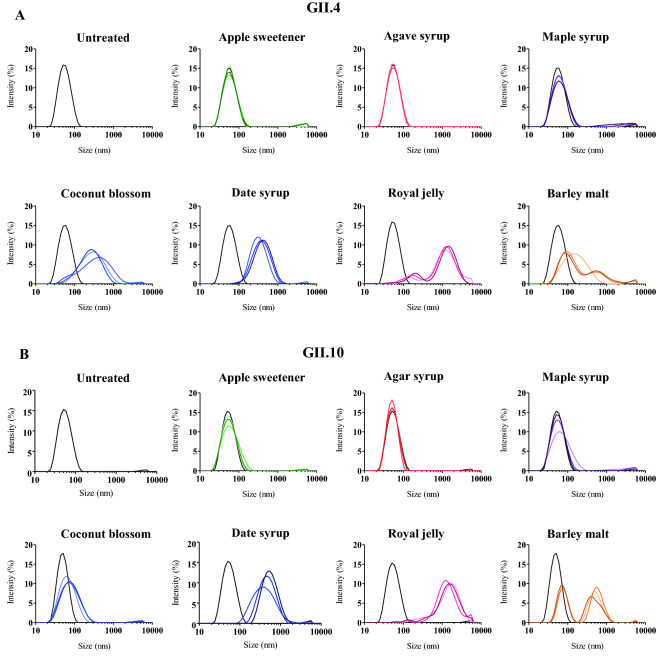
Natural extracts and DLS measurements of GII.4 and GII.10 VLPs. DLS was used to evaluate the hydrodynamic size of the VLPs (measured in intensity) after natural extract treatment (final concentration of extracts 12.5%) at 10 min, 60 min, and 120 min (represented by light, medium, dark colored lines, respectively). Untreated VLPs (black line) showed a single peak at all time points. (**A**) For GII.4, treatment with apple sweetener, agave syrup, and maple syrup there was little effect on the diameters. For coconut blossom syrup and date syrup GII.4 VLPs diameters were larger than the untreated VLPs. For royal jelly and barley malt two new GII.4 VLP peaks with increased diameters were detected. (**B**) For GII.10, apple sweetener and agave syrup treatment of VLPs did not result in increased diameters. Date syrup, coconut blossom syrup, and royal jelly increased the GII.10 VLP diameters, whereas for barley malt two peaks representing increased VLP diameters were observed. Each experiment was performed in triplicate and representative measurements were presented.

In order to evaluate how the natural extracts alter the stability of intact norovirus VLPs, the diameters of natural extract treated VLPs were measured using DLS. The untreated GII.4 and GII.10 VLPs exhibited a single peak, indicating a homogenous sample with VLP diameters of approximately 40–46 nm (Fig. 2). For GII.4, incubation with apple sweetener, agave syrup, and maple syrup indicated that the size of the VLPs were comparable with untreated VLPs. Coconut blossom, date syrup, royal jelly, and barley malt dramatically amplified the heterogeneity of GII.4 VLPs and led to an increased peak shift, indicating particle aggregation. Similarly for GII.10, apple sweetener, agave syrup, and maple syrup treatments were comparable to the untreated VLPs. Coconut blossom caused a slight increase in VLP heterogeneity, whereas date syrup, royal jelly, and barley malt amplified the heterogeneity of GII.10 VLPs and lead to an increased peak shift.

**Figure 3:**
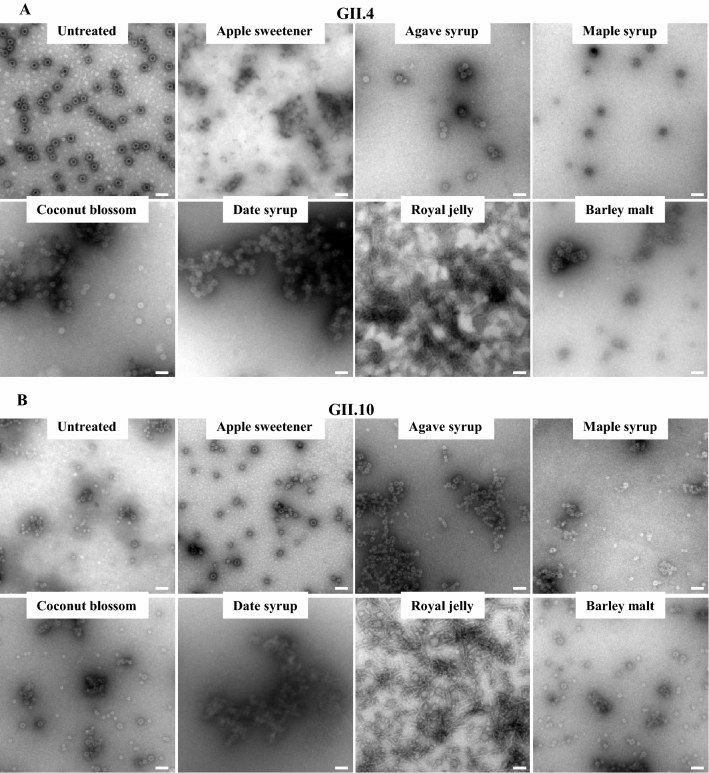
Natural extracts and EM analysis of GII.4 and GII.10 VLPs. Negative stain EM was used to evaluate the integrity of VLPs after natural extract treatment (final concentration of extracts 12.5%). With royal jelly, long rod-like shapes (from the jelly sample) obscured visualizing the treated VLPs. (**A**) Untreated GII.4 VLPs showed the typical T = 4 structure of VLPs. Agave syrup and maple syrup treated GII.4 VLPs closely resembled untreated VLPs. Treatment with apple sweetener and barley malt resulted in the formation of small aggregates. Treatment with coconut blossom syrup and date syrup led to the formation of larger GII.4 VLP aggregates. (**B**) Untreated GII.10 VLPs showed a mixture of T = 1 and T = 3 VLPs. Apple sweetener treated GII.10 VLPs closely resembled untreated VLPs. Maple syrup, coconut blossom syrup, and barley malt treated GII.10 VLPs resulted in the formation of small aggregates. GII.10 VLPs treated with agave syrup and date syrup led to formation of larger aggregates. Scale bar = 100 nm. EM analysis was performed in triplicate and different grid squares were imaged. Representative images of the samples were presented.

To directly observe the effects and compare with DLS measurements, the natural extracts were incubated with VLPs and examined using EM (Fig. 3). The morphology of the untreated GII.4 VLPs were mainly T = 4 particles, whereas the untreated GII.10 VLPs were a mixture of T = 3 and T = 1 icosahedral particles. The morphology of the GII.4 VLPs treated with apple sweetener, agave syrup and maple syrup were comparable to untreated VLPs. With coconut blossom, date syrup, and barley malt, the GII.4 VLPs were found in small and large clumps. For royal jelly, single VLPs were observed, but a large part of the EM grid contained long thin rods, which originated from the natural extract sample. Taken together, the EM results matched the DLS measurements and confirmed that several natural extracts caused VLP aggregation and clumping.

### Honey treatment

**Figure 4:**
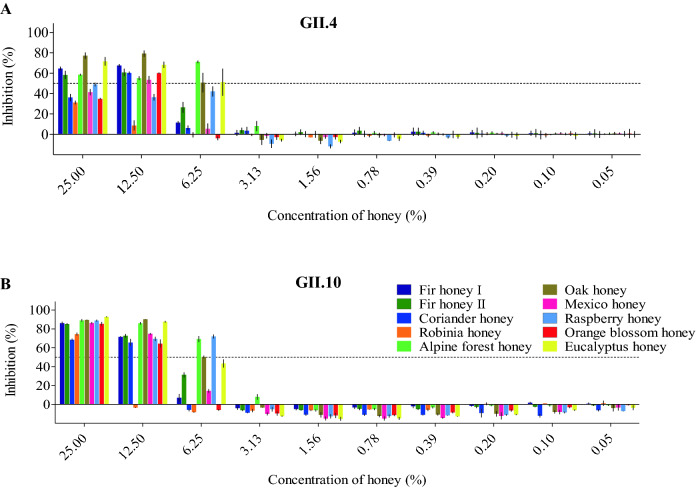
Honey samples blocking GII.4 and GII.10 VLP attachment to HBGAs. VLPs were mixed with serially diluted honey samples (fir I, fir II, coriander, Robinia, Alpine forest, Oak tree, Mexico, raspberry, Orange blossom, and eucalyptus). HBGA attachment inhibition was measured using ELISA. The final concentration of the honey samples ranged between 25 and 0.05%. All experiments were performed in triplicates and standard deviation is shown by the error bars. The IC_50_ cutoff is shown by the dashed line. (**A**) For GII.4 VLPs, raspberry honey and alpine forest honey blocked at the lowest concentrations (IC_50_ = 4.72% and 4.78%, respectively) followed by Oak tree honey (IC_50_ = 5.85%) and eucalyptus honey (IC_50_ = 6.22%). (**B**) For GII.10 VLPs, Alpine forest honey blocked at the lowest concentration (IC_50_ = 3.38%), followed by raspberry honey (IC_50_ = 5.60%), eucalyptus honey (IC_50_ = 5.96%), and Oak tree honey (IC_50_ = 6.11%).

**Figure 5:**
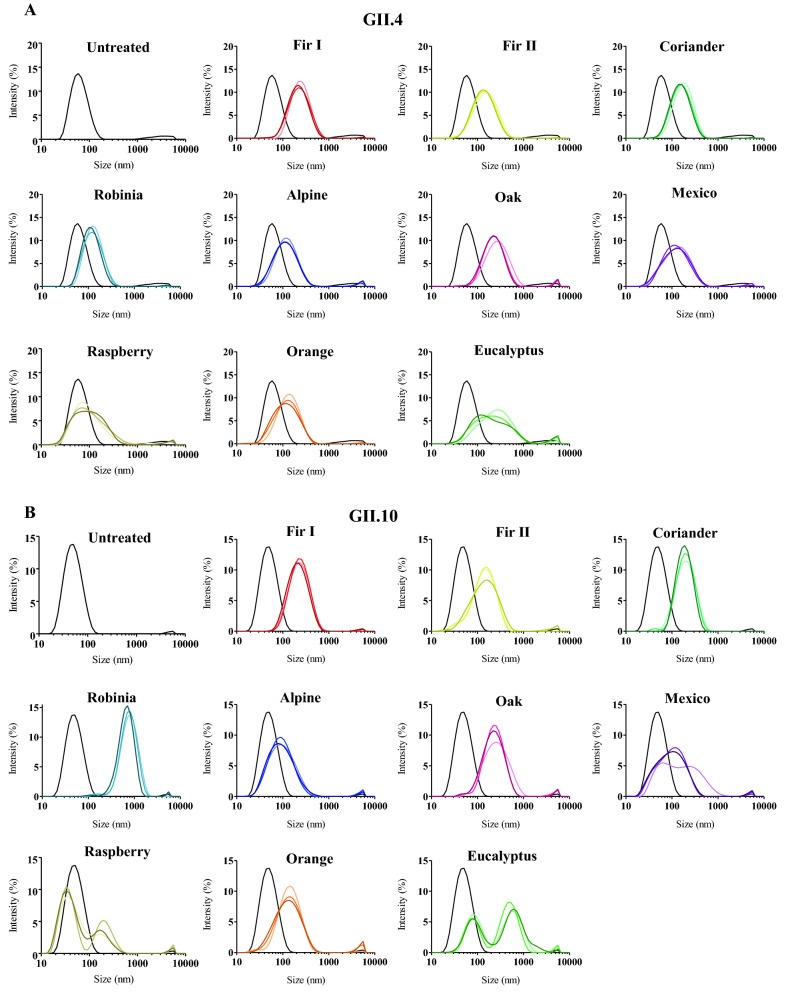
Honey samples and DLS measurements of GII.4 and GII.10 VLPs. DLS was used to evaluate the hydrodynamic size of VLPs (final concentration of honey 12.5%) at 10, 60, and 120 min (represented by light, medium and dark colored lines, respectively). For all honey samples, there was an increase in VLP diameters. (**A**) For treatment of GII.4 VLPs with fir I, fir II, coriander, Robinia, Alpine forest, Oak tree, Mexico, and orange blossom similar peak shifts were observed. Eucalyptus and raspberry treatment resulted in a flattened peak and a greater range of differently sized GII.4 VLPs aggregates. (**B**) For treatment of GII.10 VLPs, a similar pattern was observed as GII.4 VLPs, except that raspberry and eucalyptus treated GII.10 VLPs produced two peaks of increased sizes. Each experiment was performed in triplicate and representative measurements were presented.

**Figure 6:**
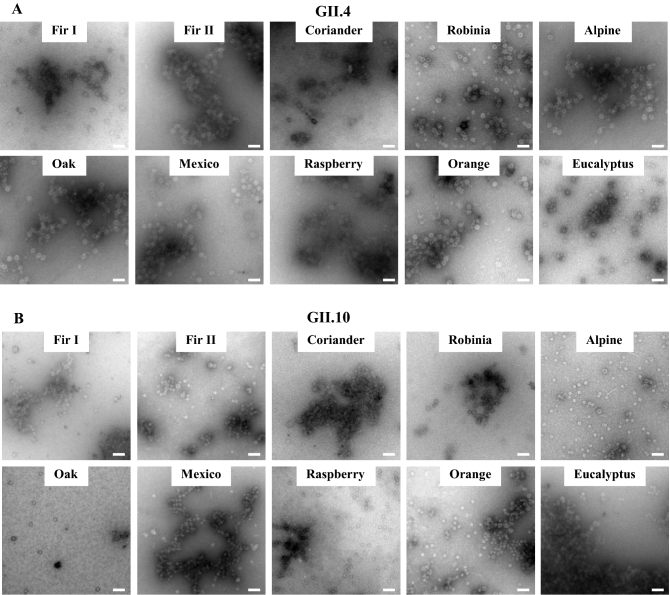
Honey samples and EM analysis of GII.4 and GII.10 VLPs. The integrity of the VLPs upon treatment with different honey samples (final concentration of honey 12.5%) was observed using EM. (**A**) For GII.4, most honey samples caused the VLPs to form small aggregates, in particular Fir II, Oak tree, and raspberry. However, single particles were also observed. (**B**) For GII.10, the VLPs treated with honey samples tended to aggregate, especially for fir I, coriander, Mexico and eucalyptus, although single particles were also found. Scale bar = 100 nm. EM analysis was performed in triplicate and different grid squares were imaged. Representative images of the samples were presented.

Ten different honey samples were examined for their ability to block norovirus GII.4 and GII.10 VLPs from binding to HBGAs (Fig. 4). The honey samples inhibited GII.4 and GII.10 VLPs at IC_50_ values ranging between 4.72–24.77% and 3.38–15.26%, respectively. For GII.4 VLPs, the honey samples with the lowest IC_50_ concentrations were raspberry honey (IC_50_ = 4.72%), Alpine forest honey (IC_50_ = 4.78%), Oak tree honey (IC_50_ = 5.85%), and eucalyptus honey (IC_50_ = 6.22%). For GII.10 VLPs, a similar finding was observed, i.e., Alpine forest honey (IC_50_ = 3.38%), raspberry honey (IC_50_ = 5.60%), eucalyptus honey (IC_50_ = 5.96%), and Oak tree honey (IC_50_ = 6.11%). Experiments with only solvent produced results similar to the PBS controls (no treatment), which indicated the solvents did not affect the inhibition. Overall, these findings showed that honey samples from different herbal and regional origins could block norovirus VLPs at varied concentrations and similarly between the two genotypes. We also evaluated the treatment with honey on the stability of VLPs using DLS (Fig. 5). All honey samples caused noticeable changes to the GII.4 and GII.10 VLP diameters, where an increase in the heterogeneity and a peak shift was observed. Treatment with Robinia honey was slightly different between the GII.4 and GII.10 VLPs, where the GII.10 had a greater increase in heterogeneity compared to GII.4 VLPs. To directly observe these effects, the honey-treated VLPs were examined using EM (Fig. 6). Most of the honey samples caused the GII.4 and GII.10 VLPs to clump into small aggregates, although single particles were also observed. This result suggested that VLPs were prone to sticking together and remaining for the most part intact.

### Propolis treatment

**Figure 7:**
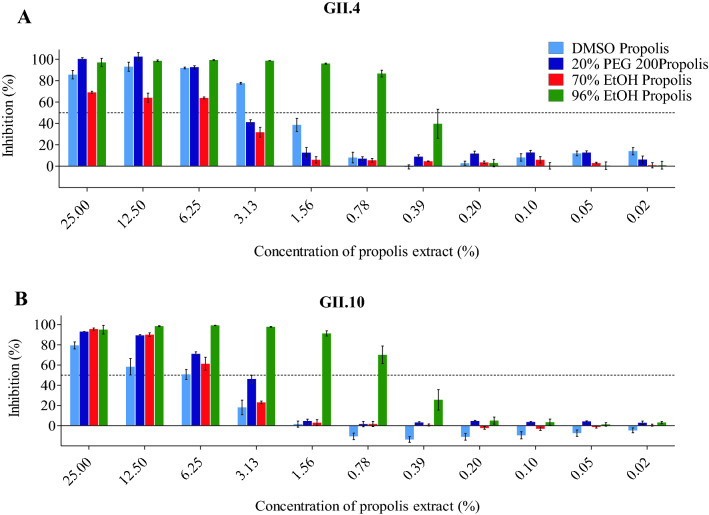
Propolis samples blocking GII.4 and GII.10 VLP attachment to HBGAs. VLPs were mixed with serially diluted propolis samples (100% DMSO propolis, 20% PEG200 propolis, 70% ethanol propolis, and 96% ethanol propolis) and HBGA attachment inhibition was measured using ELISA. The final concentration of the propolis extracts ranged between 25 and 0.02% (final concentration of solvents: DMSO 25–0.02%, PEG200 5–0.004%, 70%-ethanol 17.5–0.014%, and 90%-ethanol 24–0.0192%). All experiments were performed in triplicates and standard deviation is shown by the error bars. The IC_50_ cutoff is shown by the dashed line. Overall, all propolis samples showed a dose-dependent inhibition. (**A**) For GII.4 VLPs, the lowest concentration of inhibition was observed with 96% ethanol propolis (IC_50_ = 0.44%). (**B**) For GII.10 VLPs, the lowest concentration of inhibition was observed with 96% ethanol propolis (IC_50_ = 0.57%).

**Figure 8:**
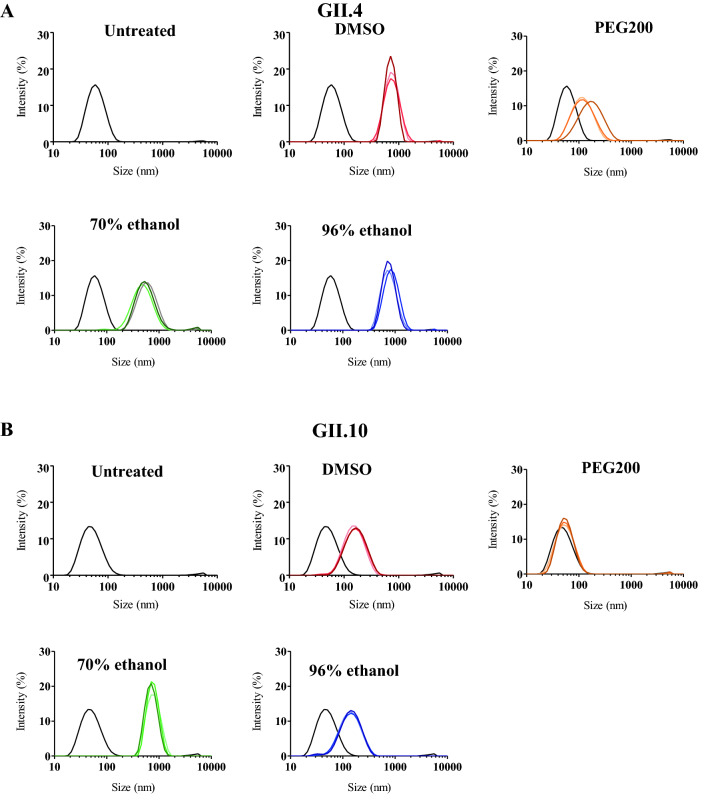
Propolis and DLS measurements of GII.4 and GII.10 VLPs. DLS was used to evaluate the hydrodynamic size of VLPs (final concentration of propolis 12.5%, final concentration of solvents: DMSO 12.5%, PEG200 2.5%, 70%-ethanol 8.75%, and 90%-ethanol 12%) at 10, 60, and 120 min (represented by light, medium and dark colored lines, respectively). Untreated VLPs showed a single peak (black lines). VLPs treated with 100% DMSO propolis, 70% ethanol propolis, and 96% ethanol propolis showed similar peak shifts for (**A**) GII.4 and (**B**) GII.10. The 20% PEG200 propolis treatment also showed a peak shift for GII.4 VLPs, although was less pronounced than with the other propolis samples, while for GII.10 VLPs, the 20% PEG200 propolis showed only a minor peak shift. Each experiment was performed in triplicate and representative measurements were presented.

**Figure 9:**
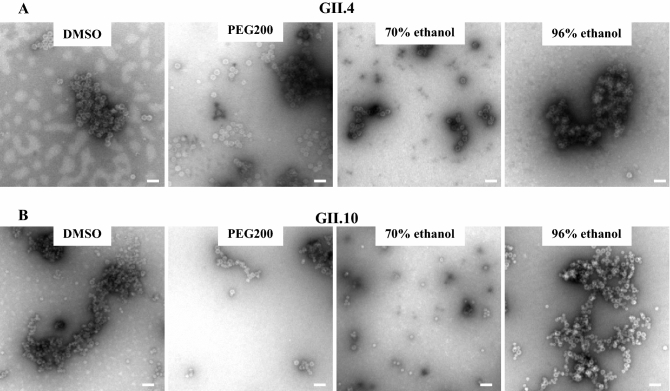
Propolis and EM analysis of GII.4 and GII.10 VLPs. Negative stain EM was used to directly determine the integrity of the VLPs. For both (**A**) GII.4 and (**B**) GII.10 VLPs, treatment with 100% DMSO propolis, 96% ethanol propolis, and 20% PEG200 propolis (final concentration of propolis 12.5%, final concentration of solvents DMSO 12.5%, PEG200 2.5%, 70%-ethanol 8.75%, and 90%-ethanol 12%) resulted in VLP aggregates, whereas mainly single VLPs were observed with 70% ethanol propolis. Scale bar = 100 nm. EM analysis was performed in triplicate and representative images of the samples were presented.

Four different propolis samples were examined for their ability to block norovirus VLPs from binding to HBGAs (Fig. 7). The propolis samples showed a dose-dependent inhibition and the GII.4 and GII.10 VLPs were blocked at low concentrations with IC_50_ values ranging between 0.44–5.38%. The highest inhibition was observed with the 96% ethanol propolis, i.e., GII.4 (IC_50_ = 0.44%) and GII.10 (IC_50_ = 0.57%). A small difference in inhibition levels was observed with DMSO propolis i.e., GII.4 (IC_50_ = 1.74%) and GII.10 (IC_50_ = 5.38%). We also evaluated propolis treatment on the stability of GII.4 and GII.10 VLPs using DLS (Fig. 8). Almost all propolis samples increased the heterogeneity and led to a peak shifts ranging between 100—1000 nm, even at short (10 min) incubation times. One propolis sample, 20% PEG200 propolis had little effect on the GII.10 VLPs, whereas for the GII.4 VLPs a shift in diameter and heterogeneity was observed. To directly observe the effects, the propolis-treated GII.4 and GII.10 VLPs were examined using EM (Fig. 9). Three of the propolis samples (DMSO, PEG200, and 70% EtOH) caused the VLPs to aggregate into clumps, although intact particles were also observed. For the 96% ethanol propolis sample, large aggregates of disassembled VLPs and very few intact particles were observed.

## Discussion

A recent study using a norovirus cell culture system, where human enteroids are used to propagate the virus, showed a norovirus-specific monoclonal antibody inhibited norovirus virions in cell culture^31^. They also observed norovirus VLP aggregation (measured using DLS) occurred when treated with the monoclonal antibody and suggested that the VLPs might cross-link which in turn sterically blocked the HBGA binding site. However, the monoclonal antibody did not bind directly to the HBGA pocket and the footprint was located on the side of the P domain. A similar observation was made with several norovirus-specific Nanobodies^5,6^. Inhibition studies using natural extracts, such as pomegranate juice, cranberry juice, and green tea extracts have indicated varied levels of HBGA inhibition, although the precise binding site on the capsid remains unknown^26–30^. The structural basis other natural samples, i.e., citrate and HMOs showed that these molecules bound precisely at the HBGA pocket on the capsid, which in turn blocked VLP attachment to HBGAs^16–19^.

Presently, there is little available for treating a norovirus infection, while vaccines and antivirals are still at the clinical trial phase and development stage, respectively. From a therapeutic point of view, most infected individuals would favor the possibility of reducing the severe symptoms, which can include profuse vomiting, nausea, fever, and diarrhea. In an outbreak setting, norovirus is highly contagious, and virions are stable in the environment for long periods. Disinfection methods include chlorine bleach and hydrogen peroxide. Alcohol based disinfectants were found to have mixed conclusions but are generally regarded as a last line for defense. For practical purposes, chlorine bleach and hydrogen peroxide generally require the closure of the contaminated areas, and this can include hospital wards, schools, cruise ships, and aged-care homes. Another important area of norovirus infections is related to food contamination, which can include oysters, clams, ice, and fruit. Contaminated food is becoming an increasing problem worldwide and decontaminating food is a high priority for the agriculture and seafood sectors. The discovery of an inhibiting, safe, and natural treatment would be of great interest and benefit. So far, decontamination methods have included UV radiation, chemical disinfectants, and high pressure^32–40^.

In the current study, we showed date syrup was the strongest inhibitor of GII.4 (IC_50_ = 0.06%) and GII.10 (IC_50_ = 0.11%) norovirus VLPs using ELISA, which was also confirmed using DLS and EM. Barley malt showed a weaker inhibition potential with GII.4 norovirus VLPs (IC_50_ = 0.90%), which was confirmed using DLS and EM, whereas for GII.10 norovirus barely malt had little effect at the tested concentrations. Honey samples were less effective at blocking GII.4 and GII.10 VLP attachment to HBGAs compared to date syrup, although treatment caused VLP aggregation. All propolis samples showed inhibition potential for GII.4 and GII.10 VLPs, especially 96% EtOH propolis (IC_50_ = 0.44% and IC_50_ = 0.57%, respectively), which was confirmed using DLS and EM. Our results were comparable to citrate and Nanobody treatments that showed the integrity of norovirus VLPs were compromised and changes in morphology included particle disassembly and aggregation^6,18^. Likewise, treatments with other natural extracts observed similar findings. For example, grape seed extract treatment resulted in particle deformation and enlargement^28^. Several studies using surrogate noroviruses (feline calicivirus and murine norovirus) also found that natural fruits or their components could inhibit or reduce infectivity^41–45^.

In summary, the data developed in this study have produced some promising findings. The idea of natural therapy against viral infections is not entirely new and is just becoming an interesting topic of noroviruses inhibition. Further studies that examine norovirus inhibition in cell culture are expected as well as testing other natural extracts are planned.

## Methods

### Natural extracts, honey, and propolis

Seven natural extracts (date syrup, barely malt, apple sweetener, agave nectar, coconut blossom syrup, royal jelly and maple syrup), ten different honey samples (Alpine forest, coriander, eucalyptus, fir I, fFir II, Mexico, Oak tree, Orange blossom, and Robinia), and two propolis samples (MF and tincture) were purchased from various sources (Table 1). Natural extracts and honey samples were diluted in PBS (pH 7.4), filtered (0.45 µm), and then stored at 4 °C. Propolis MF was mashed and then incubated in three different solvents, i.e., either 20% PEG200, 100% DMSO, or 70% ethanol. These propolis samples were left for 30 days at room temperature and then clarified by centrifugation and filtering (pore size 0.45 µm), and the final solution was stored at 4 °C. Propolis tincture was purchased ready to use in 96% ethanol. Controls with solvents (i.e., PEG200, DMSO, and ethanol) at concentrations comparable with the diluted propolis samples were performed, to ensure that inhibition or aggregation effects were not caused by solvents.

**Table 1:** Description of natural extracts, honey, and propolis samples.

Natural extracts	Company/origin
Agave nectar	BioTropic GmbH, Germany
Apple sweetener	Rigoni di Asiago, Italy
Barley malt	Lindenmeyer, Germany
Coconut blossom syrup	Rapunzel Naturkost, Germany
Date syrup	Rapunzel Naturkost, Germany
Maple syrup	Naturata, Canada
Royal jelly	Cum Natura GmbH, Germany
Honey—Alpine forest	Breitsamer Honig, Italy, Austria
Honey—Coriander	Atrium Import GmbH, Ukraine
Honey—Eucalyptus	GEPA, Uruguay
Honey—Fir I	RU 0,762,880
Honey—Fir II	Imkerei Bunsen, Black Forrest, Germany
Honey—Mexico	GEPA, Mexico
Honey—Oak tree	Imkerei Ullrich, Odenwald, Germany
Honey—Orange blossom	GEPA, Mexico
Honey—Raspberry	Imkerei Bernhard Niepalla, Germany
Honey—Robinia	Wabenschatz, Germany
Propolis MF (Fellbach)	Local beekeeper (Maile, Fellbach)
Propolis tincture 96% EtOH	Miel Company S.C., Spain

### VLP expression

Norovirus VP1 of two different GII genotypes, GII.4 (JX459908, Sydney2012) and GII.10 (AF504671, Vietnam 026), were expressed in insect cells as described previously^46^. Briefly, VLPs were clarified from insect cell medium, pelleted by ultracentrifugation, and then further purified using CsCl gradient ultracentrifugation.

### HBGA blocking assay

Blocking assays were performed using a method as descried earlier for HMOs^16,17^. Briefly, 96-well plates were coated with pig gastric mucin type III (PGM), washed three times with PBS containing 0.1% Tween 20 (PBS-T), and subsequently blocked with 5% skimmed milk in PBS. The concentration of untreated GII.4 and GII.10 VLPs were optimized for binding to PGM as previously described^17,47^. The GII.4 VLPs (1 μg/ml) or GII.10 VLPs (10 μg/ml) were mixed (1:1) with serially diluted extracts (starting at a concentration from 25%) for 3 h at room temperature. Plates were washed three times with PBS-T, then 100 µl of each VLP/ natural extract mixture was added to triplicate wells for 2 h at room temperature. After washing, 100 µl of GII.4 or GII.10 genotype-specific polyclonal rabbit antibody was added as a primary detection antibody for 1 h at room temperature. Following a wash step, horseradish peroxidase (HRP) conjugated polyclonal anti-rabbit antibody or HRP-conjugated streptavidin was added to the wells and incubated for 1 h at room temperature. All plates were washed and developed with *o*-phenylenediamine dihydrochloride (OPD) and H_2_O_2_ in the dark for 30 min at room temperature. The reaction was stopped with 3 N HCl and absorbance was measured at 490 nm (OD_490_). Negative controls, i.e., VLPs without inhibitor and no VLPs were used for all plates. The OD_490_ value of the untreated VLPs was set as the reference value corresponding to 100% binding. The concentrations of the different extracts were defined as 100% pre-dilution. The percentage of inhibition was calculated as [1-(treated VLP mean OD_490_/mean reference OD_490_)] × 100. The half-maximal inhibitory concentration (IC_50_) was determined using Prism software (version 8.0). All experiments were performed with three replicates and the mean and standard deviation calculated.

### Dynamic light scattering

The hydrodynamic diameter of VLPs was analyzed using dynamic light scattering (DLS) with a Zetasizer Nano S (Malvern). The VLPs (1 mg/ml) and natural extracts (25% from original stock) were mixed (1:1) and incubated for 10, 60, and 120 min at room temperature, diluted in 1 ml of distilled water, and then measured. For propolis samples, a 25% working solution was left for 48 h at 4 °C and then briefly centrifuged to remove precipitated beeswax. Intensity measurements were performed at 25 °C in three runs with 15 measurement cycles.

### Electron microscopy

The VLPs (treated and untreated) were analyzed using negative stain electron microscopy (EM) as described^6^. A 25% stock of natural extracts or honey were mixed (1:1) with 1 mg/ml VLPs and incubated for 1 h at room temperature (final concentration of natural extract and honey = 12.5%). Propolis samples were prepared as described for DLS. Samples were diluted 1:40 in distilled water and immediately loaded on carbon coated EM grids. Grids were washed with distilled water, stained with 0.75% uranyl acetate, and then examined on a Zeiss 900 electron microscope. Numerous EM images were analyzed for each sample and one representative was shown.

## Data Availability

The datasets generated during and/or analysed during the current study are available from the corresponding author on reasonable request.
